# Molecular Evolution of Enterovirus 68 Detected in the Philippines

**DOI:** 10.1371/journal.pone.0074221

**Published:** 2013-09-20

**Authors:** Tadatsugu Imamura, Akira Suzuki, Socorro Lupisan, Michiko Okamoto, Rapunzel Aniceto, Rutchie J. Egos, Edgardo E. Daya, Raita Tamaki, Mariko Saito, Naoko Fuji, Chandra Nath Roy, Jaime M. Opinion, Arlene V. Santo, Noel G. Macalalad, Amado Tandoc, Lydia Sombrero, Remigio Olveda, Hitoshi Oshitani

**Affiliations:** 1 Tohoku University Graduate School of Medicine, Sendai, Japan; 2 Tohoku-RITM Research Center for Emerging and Reemerging Infections, Muntinlupa City, the Philippines; 3 Eastern Visayas Regional Medical Center, Tacloban City, the Philippines; 4 Leyte Provincial Hospital, Palo City, the Philippines; 5 Tacloban City Health Catchment Center, Tacloban City, the Philippines; 6 Tanuan Rural Health Unit, Tanuan City, the Philippines; 7 Research Institute for Tropical Medicine, Muntinlupa City, the Philippines; The University of Hong Kong, China

## Abstract

**Background:**

Detection of Enterovirus 68 (EV68) has recently been increased. However, underlying evolutionary mechanism of this increasing trend is not fully understood.

**Methods:**

Nasopharyngeal swabs were collected from 5,240 patients with acute respiratory infections in the Philippines from June 2009 to December 2011. EV68 was detected by polymerase chain reaction (PCR) targeting for 5′ untranslated region (5′UTR), viral protein 1 (VP1), and VP4/VP2. Phylogenetic trees were generated using the obtained sequences.

**Results:**

Of the 5,240 tested samples, 12 EV68 positive cases were detected between August and December in 2011 (detection rate, 0.23%). The detection rate was higher among inpatients than outpatients (*p*<0.0001). Among VP1 sequences detected from 7 patients in 2011, 5 in lineage 2 were diverged from those detected in the Philippines in 2008, however, 2 in lineage 3 were not diverged from strains detected in the Philippines in 2008 but closely associated with strains detected in the United States. Combined with our previous report, EV68 occurrences were observed twice in the Philippines within the last four years.

**Conclusions:**

EV68 detections might be occurring in cyclic patterns, and viruses might have been maintained in the community while some strains might have been newly introduced.

## Introduction

Human enterovirus 68 (EV68) is a member of human enterovirus D (HEV-D), which belongs to the *Enterovirus* genus, *Picornaviridae* family. EV68 was first isolated in 1962 from 4 pediatric patients hospitalized with lower respiratory tract infection, in California [1]. Since then, EV68 has been identified only sporadically. In the enterovirus surveillance conducted in the United States from 1970 to 2005, only 26 strains were reported over 35 years [2]. However, the number of reported EV68 cases has remarkably increased in recent years [3]. Between 2008 and 2009, we detected EV68 in 21 pediatric patients who were hospitalized with severe pneumonia in the Philippines [4]. Increased EV68 detection has been reported from different parts of the world, including the United States [5], Europe [6], [7], Africa [5] and Asia [8], [9]. However, it remains elusive whether this increasing trend was due to a real increase of cases or increased detection owing to improvement in detection methods such as usage of polymerase chain reaction (PCR). Retrospective analysis of stored respiratory specimens by PCR revealed detection of EV68 every year in the Netherlands since 1996 [6] and in Yamagata, Japan since 2005 [8], with smaller number of positive cases before the increased detections in 2010 at each study site. However, it is still unknown whether those viruses detected each year had been maintained in the community or newly introduced.

Majority of reported EV68 cases were associated with acute respiratory infections including a considerable number of severe cases especially among children. A total of 5 fatal cases have been reported so far, including 2 pediatric patients hospitalized with pneumonia in the Philippines [4], [10], [11]. In one previous study among the patients with acute respiratory infections (ARI) in the Netherlands, detection rate of EV68 was significantly higher in ARI patients with bronchiolitis compared to those without it [6], which suggested that EV68 might be more frequently associated with severe clinical symptoms. In the study, EV68 detection rate was highest among patients aged 50–59 years and it was the lowest among those aged younger than 10 years. In a study conducted in Thailand, EV68 was more likely to be detected among hospitalized pediatric patients compared to children who consulted outpatient clinics [9], which concurred with the report from the Netherlands. However, the etiological significance of EV68 is yet to be determined. Therefore, the current study aimed at investigating the molecular evolution of circulating EV68 viruses in the Philippines.

## Materials and Methods

### 2.1. Sample Collection

We conducted prospective studies for respiratory viruses in Leyte island, the Philippines from June 2009 to December 2011 which include three studies; pediatric pneumonia study at Eastern Visayas Regional Medical Center (EVRMC) since May 2008, adult pneumonia study at EVRMC since May 2010 and influenza-like illness (ILI) study at outpatient clinics of Leyte Provincial Hospital (LPH), Tacloban City Health Catchment Center (TCHCC) and Tanuan Rural Health Unit (TRHU) since January 2010. Nasopharyngeal swabs were collected from patients hospitalized at EVRMC with the diagnosis of severe acute respiratory infection (SARI) as defined by the World Health Organization [12], or those who visited the outpatient clinic of LPH, TCHCC or TRHU with ILI, as defined as fever >38°C accompanied by cough or sore throat. Clinical samples were collected from 1,187 hospitalized pediatric patients from June 2009 to December 2011, 456 hospitalized adult patients from May 2010 to December 2011, and 3,597 outpatients at LPH, TCHC and TRHU from January 2010 to December 2011 (Table 1).

**Table 1:** Patient demographics of the childhood pneumonia study, adult pneumonia study, and ILI study.

	Number of participants				Age distribution(median)†	Gender distribution(% male)
	Total	Year				
		2009	2010	2011		
Childhood pneumonia	1187	143 (June∼)	344	700	4D–14Y (11M)	56.1%
Adult pneumonia	456	NC*	165 (May∼)	291	14Y–89Y (59Y)	50.4%
Influenza-like illness	3597	NC*	1663	1934	11D–82Y (2Y6M)	50.0%

* NC; Studies not conducted,

† D; days, M.; months, Y; years.

Number of participants and patient demographics of those enrolled in the 3 studies are listed.

EV68 sequences obtained from the same pediatric pneumonia study between May 2008 and May 2009 were also included in molecular analysis to define their phylogenetic and evolutionary relationship with those detected in this study. Additionally, in order to detect the mixed infections, collected samples were also tested for panels of viruses, including influenza viruses, human metapneumovirus, respiratory syncytial virus, and human rhinoviruses following our previously reported protocol [13].

After obtaining written informed consent, respiratory samples and clinical information were collected from patients or their guardians at the time of hospital admissions or outpatient consultations. The study was approved by the Ethical Committee of Tohoku University Graduate School of Medicine, Japan and the Institutional Review Board of the Research Institute for Tropical Medicine, the Philippines.

### 2.2. RT-PCR and Sequencing

RNA was extracted from clinical specimens using the QIA Viral RNA Mini kit (QIAGEN) according to the manufacturer’s instructions. cDNA was synthesized using random primers (Invitrogen) and MMLV Reverse Transcriptase (Invitrogen).

Samples were screened by reverse transcription-polymerase chain reaction (RT-PCR) targeting 5′ untranslated region (5′UTR) of Rhinovirus using primer pairs DK001 [14] and DK004 [15] (Table 2). The PCR amplicons were purified using a SUPREC-PCR kit (TaKaRa) or QIAquick PCR Purication Kit (250) (QIAGEN), and used as templates in cycle sequencing. (ABI Prism BigDye Terminator Cycle Sequencing Ready Reaction kit, version 1.1; Applied Biosystems) in an automated sequencer (Applied Biosystems; 3130/3130 xl Genetic Analyzers, 3730/3130 xl DNA Analyzers). For the samples showing high identity to previously reported EV68 in 5′UTR sequences, PCR and sequence analysis targeting VP1 and VP4/VP2 were conducted using primer pairs; 484 and 222 [16], EV68-VP1F and EV68-VP1R or 485 [16], and EV68-VP4F and EV68-VP2R (Table 2).

**Table 2:** Primers used for detection and analysis of Enterovirus 68.

Primer’s name (ref)	Primer’s sequence (5′ → 3′)	Location (Location number*)
DK001 *(14)*	CAAGCACTTCTGTTTCCC	5′UTR (164–168)
DK004 *(15)*	CACGGACACCCAAAGTAGT	5′UTR (483–501)
EV68-VP4F	GGACCCATCAAAATTCACTG	VP4 (876–895)
EV68-VP2R	CCATTGATGTGGAAATATTG	VP2 (1451–1470)
484 *(16)*	GGRTCYCAYTACAGGATGT	VP1 (2197–2215)
222 *(16)*	CICCIGGIGGIAYRWACAT	VP1 (2933–2951)
EV68-VP1F	ACCATTTACATGCAGCAGAGG	VP1 (2393–2413)
EV68-VP1R	GACAAGAACTTTTTCAAATGGACAA	VP1 (2683–2707)
485 (*16*)	ACATCTGAYTGCCARTCYAC	2A (3425–3406)

* Location number is corresponding to the genome of EV68 Fermon strain (AY426531).

### 2.3. Sequence Analysis

Sequence analysis was conducted by MEGA (version 5) software. Phylogenetic trees were generated by using neighbor-joining method, with maximum composite likelihood as a substitution model.

The divergence time of EV68 was estimated by using Bayesian Markov chain Monte Carlo (MCMC) approach implemented in the BEAST package v1.7.1. Lognormal relaxed clock (uncorrected) was used with a tree prior of coalescent Bayesian Skyline, and the Hasegawa, Kishino and Yano 1985 (HKY85) as nucleotide substitution model with gamma as site heterogeneity model. The MCMC chains were run for 10 million iterations, with subsampling at every 10000. A 10% burn-in was discarded and maximum clade credibility tree was summarized using TreeAnovator v1.7.1, with Bayesian posterior probability (BPP) values for each node. Statistical confidence for each parameter estimate was represented by the 95% highest probability density (HPD) intervals around the marginal posterior parameter means. The MCMC process was inspected by using TRACER v1.5. Maximum clade credibility tree was generated by using FigTree v1.3.1.

The strains of previously studied VP1 sequences which were used for generating Bayesian tree were listed in Table S1.

### 2.4. Patient Information

Patient information including demographics, symptoms, radiological findings, O2 saturation level (SpO2), and clinical outcomes were collected using clinical record form. The O2 saturation level was measured at the tip of first fingers or big toes using PalmSAT2500 (Nonin Medical Inc., Plymouth, MN, USA) following manufacturer’s instructions The O2 saturation level measured after the initiation of O2 treatment or giving bronchodilator was excluded from the analysis. Information collected from the patients who were positive for EV68 in 2011 was analyzed together with those collected from EV68 positive patients in 2008–09, by using JMP Pro 9.0.2 software (SAS Institute Inc., Cary, NC, USA).

## Results

### 3.1. Detection of EV68

**Figure 1:**
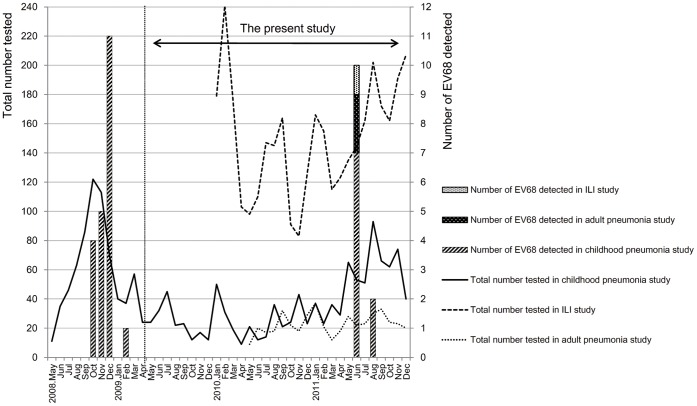
Temporal distribution of EV68 in Eastern Visayas Region, the Philippines, May 2008- December 2011. Information about the address was obtained from parents of the pediatric patients. The bar graph shows the number of EV68 cases in each month in Eastern Visayas region. The month without any bars indicates no EV68 detected cases on that month. The total number of tested samples in each of three studies is shown in line graph.

EV68 was detected in 12 samples out of 5,240 samples tested by RT-PCR targeting 5′UTR. Detected 5′UTR nucleotide sequences were deposited in GenBank with accession number of AB817717–AB817728. The positive samples showed 95.2–100% similarities to 5′UTR sequences of previously reported EV68. Notably, all 12 cases were detected from June to August 2011 (Figure 1), with 0.23% (12/5,240) detection rate. Of them, 9 cases were detected from the pediatric pneumonia study (9/1,187, 0.76%), 2 were from the adult pneumonia study (2/456, 0.44%), and 1 was from the ILI study (1/3,597, 0.028%).

### 3.2. Patients

Among a total of 33 patients (21 patients in 2008–09, and 12 patients in 2011) who were positive for EV68 in respiratory samples, the median age of the patients was 1 year and 8 months ranged from 1 month to 76 years, and 18 of them were male (54.5%).

Commonly observed symptoms among 30 hospitalized children (2008–2009; 21, 2011; 9) who were positive for EV68, included cough (100%; 30/30), chest indrawing (100%; 30/30), difficulty in breathing (83.3%; 25/30), and wheezing (60%; 18/30). Among these 30 cases, O2 saturation level was measured before initiation of oxygen treatment as well as giving bronchodilator for 19 patients, and their SpO2 value was ranged from 86% to 100% with a median value of 91%. On physical examination by clinicians, rales were found on auscultation in all 30 patients, however, abnormalities in chest X ray were found only in 8 patients including infiltration in 5 patients and both infiltration and consolidation in 3 patients.

In 2 adult patients, cough and difficulty in breathing were observed, however no wheezing was found. Rales on auscultation and infiltrates on chest radiographs were found in both patients. SpO2 levels before O2 therapy in those 2 patients were 98% and 95% respectively.

For the EV68 positive patient who was detected in ILI study, chest indrawing and rales were not found in physical examination.

Among 11 hospitalized patients, 9 were discharged and 2 died, including 1 fatal case from the pediatric pneumonia study and another from the adulthood pneumonia study. The fatal adult patient with EV68 had a comorbidity of hepatic cirrhosis.

### 3.3. Phylogenetic Analysis

**Figure 2:**
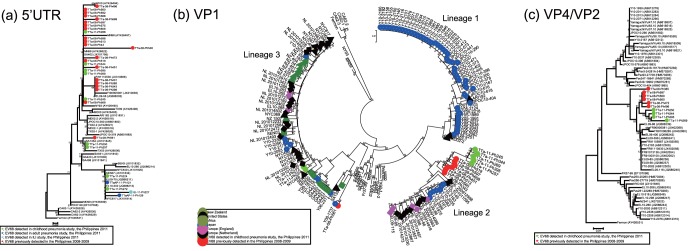
Phylogenetic tree of selected EV68 strains based on the nucleotide sequence of three genome regions. (a) the partial 5′UTR, (b) partial VP1, and (c) partial VP4/VP2. EV68 strains in this study are indicated by •. Phylogenetic analysis was performed by using nucleotide alignments and the neighbor-joining method, as implemented in MEGA software.

The viral genome of VP1 region was amplified in 7 out of the 12 samples positive for 5′UTR, and VP4/VP2 region was amplified in 4. The samples positive for VP1 and VP4/VP2 were collected from the pediatric pneumonia study. Detected VP1 and VP4/VP2 sequences were deposited in GenBank with accession number of AB817710–AB817716, and AB829882–AB829891 respectively. Phylogenetic trees were drawn based on 5′ UTR, VP1, and VP4/VP2 sequences including those detected between 2008 and 2009 in the Philippines (Figure 2).

Of the sequences obtained from 9 hospitalized children in 2011, 5 (TTa-11-Ph245, 266, 269, 344, and 395) belonged to the same cluster as those from 19 hospitalized children in the Philippines between 2008 and 2009 based on a phylogenetic tree of 5′UTR (Figure 2a). Of the remaining 4 sequences, 2 (TTa-11-Ph224 and 272) formed a distinct lineage together with sequences obtained from 2 adult hospitalized patients (TTaAP-11-Ph121 and 129) and 1 ILI patient (ILI-11-Ph227) in the present study. This lineage also included sequences detected from Senegal, England, and New York. The remaining 2 (TTa-11-Ph250 and 257) belonged to the cluster including previously reported sequences from the United States (TX03), the Philippines (TTa-08-Ph561) and South Africa (SA1354) (Figure 2a).

Among 12 patients who were positive for EV68 in respiratory specimens collected in 2011 (9 from the childhood pneumonia study, 2 from the adult pneumonia study, and 1 from the ILI study), VP1 sequences were amplified only for 7 samples from childhood pneumonia study. On the phylogenetic tree based on VP1 sequences, 5 out of 7 sequences detected in 2011 were in the same lineage; lineage 2, along with sequences detected from the Philippines in 2008 (TTa-08-Ph343, 451, 513, 519, 560, 597, and 608), Yamagata; Japan in 2010, the Netherlands in 2009 and 2010, and England in 2009 (Figure 2b). Lineage 2 included a branch that was exclusively consisted of sequences from the Philippines, which included 7 out of 8 strains in 2008 and 5 out of 7 strains in 2011. The sequences in this branch had high homologies of 97.4–99.9% and formed a clearly distinct cluster from other sequences in lineage 2, with high bootstrap value of 82 at the node. Within lineage 2, none of the strains detected in the Philippines had more than 98.5% homology with EV68 sequences reported from other countries.

Among 7 EV68 strains identified in 2011, 2 (TTa-11-Ph224 and 272) were grouped in lineage 3 which also included the strain identified in the Philippines in 2008 (TTa-08-Ph561) (Figure 2b). EV68 in lineage 3 shared a unique characteristic of 3 nucleotide deletions at the positions 2806–2808 in the VP1 region compared to the Fermon strain (the prototype strain of EV68), which resulted in codon deletions [16]. Although 3 strains from the Philippines shared the same branch, those identified in 2011 were clustered in a different branch from the strain in 2008 (Figure 2b).

One of the strains detected in the Philippines in 2011 (TTa-11-Ph257) belonged to lineage 2 on the phylogenetic tree based on VP1 sequences, however, its 5′UTR sequences were closely related to previously reported sequences including TX03, SA1354 and TTa-08-Ph561 (Figure 2a). Sequences of these 3 strains (TX03, SA1354 and TTa-08-Ph561) belonged to lineage 3 and formed one cluster together with those detected in Yamagata, Japan in 2006 and 2008 (Y06-2311 and Y08-1737) on the phylogenetic tree for VP1.

Another strain detected in the Philippines in 2011 (TTa-11-Ph250) also belonged to the same cluster with TTa-08-Ph561 on the phylogenetic tree based on 5′UTR sequences, however, its VP1 sequences were not amplified. Strains detected in adult pneumonia study (TTaAP-11-Ph121 and 129) and ILI study (ILI-11-Ph227) formed a branch together with other strains including those identified in childhood pneumonia study in 2011 (TTa-11-Ph224 and 272) on the 5′UTR tree, however, VP1 sequences of these 3 strains were also not amplified.

On the phylogenetic tree based on VP4/VP2 sequences, sequences detected in the Philippines formed one cluster together with those detected in England in 2009, France in 2009, and Yamagata, Japan in 2010 (Figure 2c). Among the sequences detected in the Philippines, those detected in 2011 formed one distinct lineage, and 5 (TTa-08-Ph472, 496, 560, 569, and 597) out of 6 sequences detected in 2008 formed another distinct branch. Total of 5 strains from the Philippines (TTa-08-Ph560, TTa-08-Ph597, TTa-11-Ph269, TTa-11-Ph344, and TTa-11-Ph395) belonged to lineage 2 on the phylogenetic tree based on VP1 sequences, and those strains were classified into the same cluster on the phylogenetic tree for VP4/VP2 region. The sequences from Yamagata (Y10-2082 and Y10-2086) and England (EL09-56, EL09-85, EL09-86, EL09-116 and EL09-398) belonged to the lineage 2 on the VP1 tree and also belonged to the same cluster on the VP4/VP2 tree. The strains from New York, the United States (NYC403), England (EL10-260, 268, 298, and 318), New Zealand (NZ541), Yamagata, Japan (Y10-2076, 2256, and 2336) belonged to lineage 3 on the phylogenetic tree based on the VP1 sequences, and belonged to the same cluster on the phylogenetic tree for VP4/VP2 region (Figure 2c). On the phylogenetic tree for VP1, 3 strains from the Philippines (TTa-08-Ph561, TTa-11-Ph224, and TTa-11-Ph272) belonged to the lineage 3 together with those strains from other countries. However, the VP4/VP2 sequences of these 3 strains from the Philippines were not amplified.

### 3.4. Bayesian Tree Analysis

**Figure 3:**
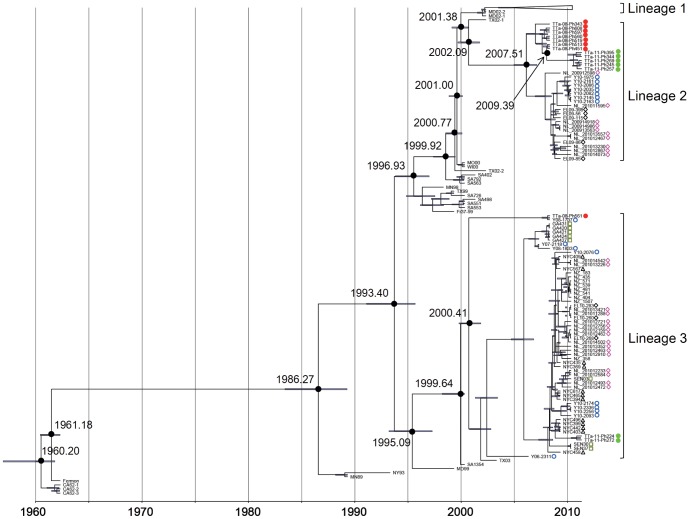
Bayesian evolutionary tree of selected EV68 strains based on the nucleotide sequence of partial VP1. EV68 strains detected in the present study are indicated by •. Bayesian tree was generated by BEAST software.

The Bayesian evolutionary tree was generated, in which VP1 sequences of EV68 detected in recent years formed 3 distinct lineages. Minor differences were observed in the estimated divergence time of each node compared to the previous report [9] which might be due to the differences in the data set used for analysis. Among 7 sequences obtained from pediatric pneumonia patients in the Philippine in 2011, 5 (TTa-11-Ph245, 257, 269, 344, and 395) formed a distinct cluster in lineage 2 and the remaining 2 (TTa-11-Ph224 and 272) formed a cluster in lineage 3 (Figure 3). In the Bayesian tree, lineages 1 and 2 were diverged from the common ancestor, approximately at the estimated node age of 2001 (2001.14–2001.97 of 95% HPD with median of 2001.38). At the estimated node age of 2007 (2005.90–2008.92 of 95% HPD with median of 2007.51), lineage 2 further diverged into two clusters, one of which exclusively consisted of sequences from the Philippines. In lineage 2, the Philippines-2011 cluster was diverged from the Philippines-2008 cluster in 2009 (2009.16–2009.80 of 95% HPD with median of 2009.39). In the other clusters of lineage 2 which did not include any sequences detected from the Philippines, consisted of those detected in Yamagata; Japan, England and the Netherlands. All the sequences detected in Yamagata formed a distinct cluster which diverged from other sequences detected in European countries.

Sequences of the remaining 2 cases from the Philippines in 2011 (TTa-11-Ph224 and TTa-11-Ph272) belonged to lineage 3 together with sequences including those detected in the Philippines in 2008 (TTa-08-Ph561). Lineage 3 consisted of sequences detected from different parts of the world including Japan, the Philippines, New Zealand, the United States of America, the Netherlands, England, Gambia, and Senegal, and each cluster in this lineage did not correlate with the specific geographical distribution. TTa-11-Ph224 and TTa-11-Ph272 formed a distinct lineage which diverged from the cluster consisted of sequences detected from New York in 2009. Based on this tree, TTa-08-561 detected in the Philippines in 2008 was not likely to be ancestor to TTa-11-Ph224 and TTa-11-Ph272. In lineage 3, sequences diverged into two clusters in 2000 (2000.40–2002.18 of 95% HPD with median of 2000.41), and one of them was consisted of only 2 sequences; TTa-08-Ph561, and Y08-1737, the latter of which was detected in Yamagata, Japan in 2008.

## Discussion

We previously reported the detection of EV68 from 21 pediatric pneumonia patients in Leyte island, the Philippines between 2008 and 2009 [4]. In this study, we detected EV68 at the same study site between June and August 2011. Even though the overall detection rate was low (0.23%), all cases were detected over a period of 3 months. Moreover, the detection rate among hospitalized cases was higher (9/1,187, 0.76% of pediatric cases and 2/456, 0.44% of adult cases) than outpatients (1/3,597, 0.028%) (*p≤*0.0001), suggesting that EV68 was more likely to be associated with severe respiratory infections. The hospitalized patients positive for EV68 presented severe respiratory symptoms, and among pediatric patients, the SPO_2_ values were considerably lower than the reference value (95%–100%) of healthy infants and children [17]. Furthermore, unlike the previous study from Thailand, interestingly we did not detect any mixed infection with aforementioned viruses in our clinical samples [9].

Combined with our previous report, increased detections of EV68 were observed twice during the 4 year study period, with an interval of approximately 2 years, and no positive cases were detected between those two periods with EV68 detections (Figure 1). Possibly, EV68 recurrence follows a cyclic pattern as suggested by the retrospective analysis of stored samples in Japan and the Netherlands [6], [8]. However, in these studies, a small number of cases were detected even between periods with increased EV68 detections.

In 2008 and 2009, majority of the detected EV68 belonged to lineage 2, and only one strain was classified into lineage 3 on the phylogenetic tree based on VP1 sequences. In 2011, most of the detected EV68 shared high similarities in VP1 sequences and also classified into lineage 2, however, two strains of lineage 3 were also detected. These results indicated that EV68 of lineages 2 and 3 were co-circulating in the study area during both 2008–09 and 2011. In the VP1 amino acid sequences detected from the Philippines, nonsynonymous substitutions were commonly found in BC- and DE-loop regions, and unique amino acid sequences in those regions were associated with the categorization of detected strains into different lineages (Figure S1), which was in line with the previous report from Thailand [9]. It was previously reported that genotype replacement is associated with occurrence of Enterovirus 71 (EV71) outbreaks [18]. In our study, such replacement of EV68 lineages was not observed between two periods of increased detection, therefore, the mechanism for increased detections is still unclear. Observation of longer period is required to reveal the relationship between circulating lineages and increased detections of EV68.

One of the strains detected in the Philippines in 2011 (TTa-11-Ph257) belonged to lineage 2 on the phylogenetic tree based on VP1 sequences, however, its 5′UTR sequences were closely related to strains whose VP1 sequences were classified as lineage 3 (TX03, SA1354 and TTa-08-Ph561). This variation may be due to the recombination which is common among members of *Picornaviridae* family [19], [20]. However, we could not make any conclusions since we only amplified partial genome of 5′UTR and VP1 of this strain. The role of recombination in evolution of EV68 is yet to be defined.

Among EV68 detected in the study, 5 in lineage 2 formed a cluster which diverged from the cluster of EV68 detected in 2008–09 in the Philippines, on the Bayesian tree (Figure 3). However, 2 strains from the Philippines which belonged to lineage 3 were not diverged from viruses detected in the Philippines in 2008 and they were more closely related to viruses found in New York, the United States. This result suggests that these strains might have been newly introduced into the community. For other respiratory viruses, strains migration at global level is well documented. For examples, molecular epidemiology of respiratory syncytial virus (RSV) indicated that specific genotype BA of subgroup B has widely spread to different parts of the world [21], [22], [23]. Despite the geographical distance between New York and Leyte Island, the Philippines, it is still possible that EV68 transmission had occurred at global level. However, number of analyzed sequences in lineage 3 was too small to draw any conclusion.

In conclusion, despite the low detection rate, our study indicated etiological importance of EV68 as a potential causative agent of severe respiratory infections. In addition, increased detections of EV68 in one area were identified twice with a 2-year interval, which suggests that viruses might have been maintained in the community. Given the fact that most of the detected sequences in 2011 were closely associated with those detected in 2008, cyclic patterns of EV68 detections might be due to the accumulation of susceptible population in the community. Our study also indicates that transmission of EV68 at global level and introduction of new strains into the community might be an alternative mechanism for increased detections of EV68. Continuous monitoring is thus required to reveal the etiological importance of EV68 and the mechanisms of its emergence in cyclic patterns in the community.

## Supporting Information

Figure S1 **Identities in the amino acid sequences of VP1 detected in the Philippines.** The amino acid sequences of VP1 region detected from the Philippines were aligned in reference to the protostrain; Fermon (AF081348). The codon positions where the Philippines sequences had identical amino acid residue to Fermon strain was indicated with dots. The BC- and DE-loop regions were indicated in boxes of dotted lines.
(EPS)

Table S1 **The strains whose VP1 sequences were used for analysis.** A total of 171 strains, including 15 from the Philippines were used for molecular analysis. The collection year, location, and the accession numbers were obtained from GenBank.
(DOCX)
