# Supplementary material for: Molecular Evolution of Enterovirus 68 Detected in the Philippines
Source: PLoS One. 2013 Sep 20;8(9):e74221. doi: 10.1371/journal.pone.0074221 (PMC3779236; doi:10.1371/journal.pone.0074221)
Supplement: Table S1 — The strains whose VP1 sequences were used for analysis. A total of 171 strains, including 15 from the Philippines were used for molecular analysis. The collection year, location, and the accession numbers were obtained from GenBank. (DOCX) [file pone.0074221.s002.docx]

Table S1. The strains whose VP1 sequences were used for analysis

| Strain | Year | Location | Accession Number |
| --- | --- | --- | --- |
| Fermon | 1962 | California, the U.S. | AY426531 |
| CA62-1 | 1962 | California, the U.S. | AY426486 |
| CA62-2 | 1962 | California, the U.S. | AY426487 |
| CA62-3 | 1962 | California, the U.S. | AY426488 |
| MN89 | 1989 | Minnesota, The U.S. | AY426489 |
| NY93 | 1993 | New York, The U.S. | AY426490 |
| MN98 | 1998 | Minnesota, The U.S. | AY426497 |
| MD99 | 1999 | Maryland, The U.S. | AY426499 |
| TX99 | 1999 | Texas, The U.S. | AY426498 |
| WI00 | 2000 | Wisconsin, The U.S. | AY426494 |
| MO00 | 2000 | Missouri, The U.S. | AY426493 |
| TX02-1 | 2002 | Texas, The U.S. | AY426495 |
| TX02-2 | 2002 | Texas, The U.S. | AY426496 |
| MD02-1 | 2002 | Maryland, The U.S. | AY426491 |
| MD02-2 | 2002 | Maryland, The U.S. | AY426492 |
| TX03 | 2003 | Texas, The U.S. | AY426500 |
| NYC369 | 2009 | New York, The U.S. | JX101803 |
| NYC394 | 2009 | New York, The U.S. | JX101804 |
| NYC399 | 2009 | New York, The U.S. | JX101805 |
| NYC403 | 2009 | New York, The U.S. | JX101806 |
| NYC409 | 2009 | New York, The U.S. | JX101808 |
| NYC435 | 2009 | New York, The U.S. | JX101809 |
| NYC442 | 2009 | New York, The U.S. | JX101807 |
| NYC458 | 2009 | New York, The U.S. | JX101810 |
| NYC465 | 2009 | New York, The U.S. | JX101811 |
| NYC496 | 2009 | New York, The U.S. | JX101812 |
| NYC567 | 2009 | New York, The U.S. | JX101813 |
| NYC817 | 2009 | New York, The U.S. | JX101814 |
| ARI192 | 2009 | Arizona, The U.S. | JX101786 |
| TTa-08-Ph343 | 2008 | Tacloban, the Philippines | AB817702 |
| TTa-08-Ph451 | 2008 | Tacloban, the Philippines | AB817703 |
| TTa-08-Ph513 | 2008 | Tacloban, the Philippines | AB817704 |
| TTa-08-Ph519 | 2008 | Tacloban, the Philippines | AB817705 |
| TTa-08-Ph560 | 2008 | Tacloban, the Philippines | AB817706 |
| TTa-08-Ph561 | 2008 | Tacloban, the Philippines | AB817707 |
| TTa-08-Ph597 | 2008 | Tacloban, the Philippines | AB817708 |
| TTa-08-Ph608 | 2008 | Tacloban, the Philippines | AB817709 |
| Y05-1989 | 2005 | Yamagata, Japan | AB667885 |
| Y05-1991 | 2005 | Yamagata, Japan | AB667886 |
| Y05-2037 | 2005 | Yamagata, Japan | AB667887 |
| Y05-2038 | 2005 | Yamagata, Japan | AB667888 |
| Y05-2043 | 2005 | Yamagata, Japan | AB667889 |
| Y05-2050 | 2005 | Yamagata, Japan | AB667890 |
| Y05-2062 | 2005 | Yamagata, Japan | AB667891 |
| Y05-2124 | 2005 | Yamagata, Japan | AB667892 |
| Y05-2218 | 2005 | Yamagata, Japan | AB667893 |
| Y05-2251 | 2005 | Yamagata, Japan | AB667894 |
| Y06-2311 | 2006 | Yamagata, Japan | AB667896 |
| Y07-1703 | 2007 | Yamagata, Japan | AB667895 |
| Y07-2118 | 2007 | Yamagata, Japan | AB667897 |
| Y08-1737 | 2008 | Yamagata, Japan | AB667899 |
| Y08-1833 | 2008 | Yamagata, Japan | AB667898 |
| Y10-1939 | 2010 | Yamagata, Japan | AB614406 |
| Y10-1946 | 2010 | Yamagata, Japan | AB614408 |
| Y10-1975 | 2010 | Yamagata, Japan | AB614409 |
| Y10-1976 | 2010 | Yamagata, Japan | AB614410 |
| Y10-1980 | 2010 | Yamagata, Japan | AB614411 |
| Y10-1981 | 2010 | Yamagata, Japan | AB614412 |
| Y10-1989 | 2010 | Yamagata, Japan | AB614413 |
| Y10-2011 | 2010 | Yamagata, Japan | AB614423 |
| Y10-2013 | 2010 | Yamagata, Japan | AB614407 |
| Y10-2015 | 2010 | Yamagata, Japan | AB614424 |
| Y10-2016 | 2010 | Yamagata, Japan | AB614425 |
| Y10-2032 | 2010 | Yamagata, Japan | AB614426 |
| Y10-2034 | 2010 | Yamagata, Japan | AB614414 |
| Y10-2035 | 2010 | Yamagata, Japan | AB614427 |
| Y10-2037 | 2010 | Yamagata, Japan | AB614428 |
| Y10-2052 | 2010 | Yamagata, Japan | AB614429 |
| Y10-2070 | 2010 | Yamagata, Japan | AB614416 |
| Y10-2071 | 2010 | Yamagata, Japan | AB614417 |
| Y10-2076 | 2010 | Yamagata, Japan | AB614440 |
| Y10-2079 | 2010 | Yamagata, Japan | AB614418 |
| Y10-2082 | 2010 | Yamagata, Japan | AB614430 |
| Y10-2086 | 2010 | Yamagata, Japan | AB614433 |
| Y10-2093 | 2010 | Yamagata, Japan | AB614441 |
| Y10-2101 | 2010 | Yamagata, Japan | AB614434 |
| Y10-2116 | 2010 | Yamagata, Japan | AB614435 |
| Y10-2145 | 2010 | Yamagata, Japan | AB614431 |
| Y10-2146 | 2010 | Yamagata, Japan | AB614436 |
| Y10-2150 | 2010 | Yamagata, Japan | AB614419 |
| Y10-2155 | 2010 | Yamagata, Japan | AB614420 |
| Y10-2158 | 2010 | Yamagata, Japan | AB614421 |
| Y10-2161 | 2010 | Yamagata, Japan | AB614437 |
| Y10-2163 | 2010 | Yamagata, Japan | AB614432 |
| Y10-2166 | 2010 | Yamagata, Japan | AB614438 |
| Y10-2167 | 2010 | Yamagata, Japan | AB614439 |
| Y10-2174 | 2010 | Yamagata, Japan | AB614442 |
| Y10-2192 | 2010 | Yamagata, Japan | AB614422 |
| Y10-2256 | 2010 | Yamagata, Japan | AB614443 |
| Y10-2336 | 2010 | Yamagata, Japan | AB614444 |
| JPOC10-200 | 2010 | Osaka, Japan | AB601872 |
| JPOC10-290 | 2010 | Osaka, Japan | AB601882 |
| JPOC10-373 | 2010 | Osaka, Japan | AB601873 |
| JPOC10-378 | 2010 | Osaka, Japan | AB601883 |
| JPOC10-396 | 2010 | Osaka, Japan | AB601884 |
| JPOC10-402 | 2010 | Osaka, Japan | AB601874 |
| JPOC10-404 | 2010 | Osaka, Japan | AB601885 |
| JPOC10-412 | 2010 | Osaka, Japan | AB601875 |
| JPOC10-441 | 2010 | Osaka, Japan | AB601876 |
| JPOC10-445 | 2010 | Osaka, Japan | AB601877 |
| JPOC10-471 | 2010 | Osaka, Japan | AB601878 |
| JPOC10-573 | 2010 | Osaka, Japan | AB601879 |
| JPOC10-616 | 2010 | Osaka, Japan | AB601880 |
| JPOC10-618 | 2010 | Osaka, Japan | AB601881 |
| Fr37-99 | 1999 | France | EF107098 |
| NL-200912598 | 2009 | Netherlands | JF896311 |
| NL-200913563 | 2009 | Netherlands | JF896308 |
| NL-200914918 | 2009 | Netherlands | JF896310 |
| NL-200914986 | 2009 | Netherlands | JF896309 |
| NL-201011288 | 2010 | Netherlands | JF896297 |
| NL-201011595 | 2010 | Netherlands | JF896304 |
| NL-201012159 | 2010 | Netherlands | JF896293 |
| NL-201012233 | 2010 | Netherlands | JF896298 |
| NL-201012462 | 2010 | Netherlands | JF896291 |
| NL-201012463 | 2010 | Netherlands | JF896296 |
| NL-201012467 | 2010 | Netherlands | JF896303 |
| NL-201012472 | 2010 | Netherlands | JF896300 |
| NL-201012493 | 2010 | Netherlands | JF896301 |
| NL-201012584 | 2010 | Netherlands | JF896299 |
| NL-201012721 | 2010 | Netherlands | JF896294 |
| NL-201012756 | 2010 | Netherlands | JF896295 |
| NL-201012867 | 2010 | Netherlands | JF896307 |
| NL-201012910 | 2010 | Netherlands | JF896290 |
| NL-201013226 | 2010 | Netherlands | JF896287 |
| NL-201013230 | 2010 | Netherlands | JF896306 |
| NL-201013352 | 2010 | Netherlands | JF896289 |
| NL-201013421 | 2010 | Netherlands | JF896312 |
| NL-201013557 | 2010 | Netherlands | JF896302 |
| NL-201014073 | 2010 | Netherlands | JF896305 |
| NL-201014502 | 2010 | Netherlands | JF896292 |
| NL-201014542 | 2010 | Netherlands | JF896288 |
| EL09-56 | 2009 | England | JQ586221 |
| EL09-85 | 2009 | England | JQ586222 |
| EL09-86 | 2009 | England | JQ586223 |
| EL09-115 | 2009 | England | JQ586224 |
| EL09-398 | 2009 | England | JQ586226 |
| EL10-260 | 2010 | England | JQ586230 |
| EL10-268 | 2010 | England | JQ586231 |
| EL10-283 | 2010 | England | JQ586232 |
| NZ-183 | 2010 | New Zealand | JQ713905 |
| NZ-358 | 2010 | New Zealand | JQ713906 |
| NZ-404 | 2010 | New Zealand | JQ713907 |
| NZ-435 | 2010 | New Zealand | JQ713909 |
| NZ-481 | 2010 | New Zealand | JQ713910 |
| NZ-539 | 2010 | New Zealand | JQ713911 |
| NZ-541 | 2010 | New Zealand | JQ713912 |
| NZ-571 | 2010 | New Zealand | JQ713913 |
| NZ-1507 | 2010 | New Zealand | JQ713904 |
| SA551 | 2000 | South Africa | JX101797 |
| SA553 | 2000 | South Africa | JX101798 |
| SA563 | 2000 | South Africa | JX101799 |
| SA726 | 2000 | South Africa | JX101801 |
| SA792 | 2000 | South Africa | JX101800 |
| SA1354 | 2000 | South Africa | JX101802 |
| SA402 | 2001 | South Africa | JX101795 |
| SA498 | 2001 | South Africa | JX101796 |
| GA420 | 2008 | Gambia | JX101790 |
| GA421 | 2008 | Gambia | JX101791 |
| GA424 | 2008 | Gambia | JX101792 |
| GA427 | 2008 | Gambia | JX101793 |
| GA431 | 2008 | Gambia | JX101794 |
| SEN03 | 2010 | Senegal | JX101787 |
| SEN30 | 2010 | Senegal | JX101788 |
| SEN37 | 2010 | Senegal | JX101789 |
|  |  |  |  |

A total of 171 strains, including 15 from the Philippines were used for molecular analysis. The collection year, location, and the accession numbers were obtained from GenBank.
